# MPO–ANCA-Positive Granulomatosis with Polyangiitis with Rapidly Progressive Glomerulonephritis and Saddle-Nose Deformity: A Case Report

**DOI:** 10.3390/antib11020033

**Published:** 2022-05-09

**Authors:** Dimitra Petrou, Minas Karagiannis, Petros Nikolopoulos, George Liapis, Sophia Lionaki

**Affiliations:** 1 Department of Nephrology, National and Kapodistrian University of Athens, Attikon Hospital, 12462 Athens, Greece; dimitra.petrou90@gmail.com (D.P.); minaskar64@gmail.com (M.K.); nikolopoulospetros@gmail.com (P.N.); 2 Department of Pathology, National and Kapodistrian University of Athens, Laiko Hospital, 11527 Athens, Greece; gliapis@gmail.com

**Keywords:** vasculitis, ANCA, kidney, ear nose thorat involvement

## Abstract

Early diagnosis and initiation of appropriate immunosuppressive treatment remain the cornerstone of antineutrophil cytoplasmic antibody (ANCA)-associated vasculitis at the cost of significant toxicity. In this report, we present a case of a 69-year-old female who presented with advanced renal insufficiency and evidence of pulmonary hemorrhage and was MPO–ANCA-positive with a clinical phenotype of granulomatosis with polyangiitis. Organ involvement included rapidly progressive glomerulonephritis (GN), along with extrarenal manifestations (skin, upper and lower respiratory system involvement, and onset of saddle-nose deformity). Kidney biopsy established the diagnosis of pauci-immune crescentic, sclerotic GN. She received therapy with glucocorticoids and cyclophosphamide, mainly due to life-threatening extra-renal manifestations, such as pulmonary hemorrhage. She avoided vasculitis-related death but she developed severe therapy-related toxicity, resulting in the discontinuation of immunosuppressive therapy. Continuous re-evaluation of patients with ANCA-associated vasculitis in terms of response to immunosuppressive therapy and treatment-related toxicity is crucial for their management.

## 1. Introduction

Rapidly progressive glomerulonephritis (GN) is characterized by crescent formation in >50% of glomeruli in renal biopsy and typically progresses to end-stage kidney disease over a period of weeks to months if not treated [1]. Pauci-immune rapidly progressive GN represents the vast majority of cases, while antineutrophil cytoplasm antibodies (ANCAs) are detected in the circulation of most of these patients [2]. Early diagnosis and initiation of the appropriate immunosuppressive treatment remain the cornerstone of therapy for such patients, in order to avoid life-threatening phenomena and minimize the degree of irreversible injury. Glucocorticoid-, cyclophosphamide-, and rituximab-based regimens are the most usually used treatment options [3,4,5,6,7]. However, they are all associated with significant toxicity, i.e., infective adverse effects, which are more frequent and/or serious in patients with severely impaired renal function or dialysis-dependent patients [8]. As a result, the selection of an appropriate therapeutic regimen and duration of immunosuppressive therapy is not a non-concrete, black-or-white decision but is based on constant re-evaluation of a patient’s responsiveness and possible adverse effects, on top of age and comorbidities.

## 2. Case Description

**Figure 1:**
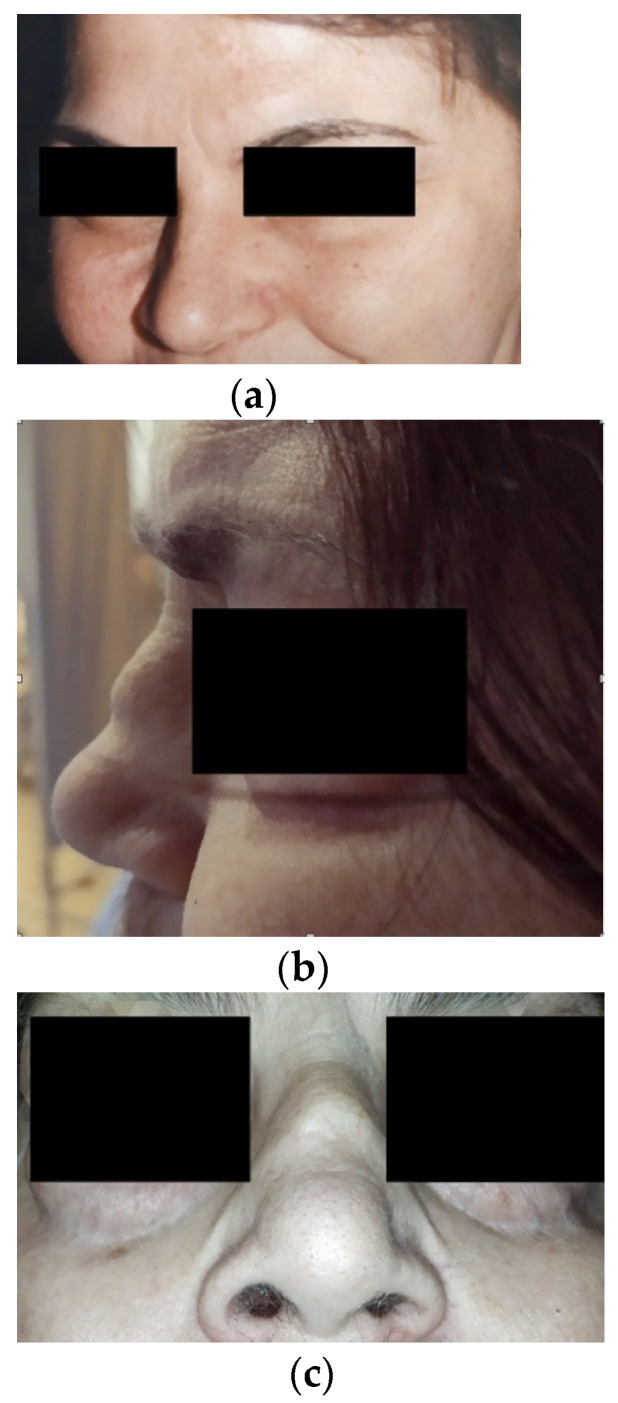
(**a**) A picture of the patients without saddle-nose deformity five years prior to presentation; (**b**,**c**) saddle-nose deformity upon presentation.

A 69-year-old female presented to the emergency department due to acute renal dysfunction, (serum creatinine: 5.5 mg/dL, serum urea 147 mg/dL), microscopic hematuria of glomerular origin (45–50 RBC/hpf), new-onset non-nephrotic proteinuria and anemia (Hb 9 g/dL). Her past medical history was significant for metabolic syndrome, i.e., obesity, type II diabetes, hypertension, and dyslipidemia for almost 20 years, along with hypothyroidism, mild ulcerative colitis, ischemic stroke due to interior carotid artery thrombosis, thrombophilia due to double heterozygous of FII-FV, pulmonary embolism due to deep vein thrombosis, smoking (50 pack-years). Prior to admission, she was on amlodipine, metoprolol, allopurinol, atorvastatin, levothyroxine, rivaroxaban, folic acid, omeprazole, metformin, and gliclazide. Physical examination revealed lower extremity edema, necrotic lesions of the skin in upper extremities, and nasal ridge caving (Figure 1), which was developed within the past few weeks according to the patient’s report (Figure S1).

**Figure 2:**
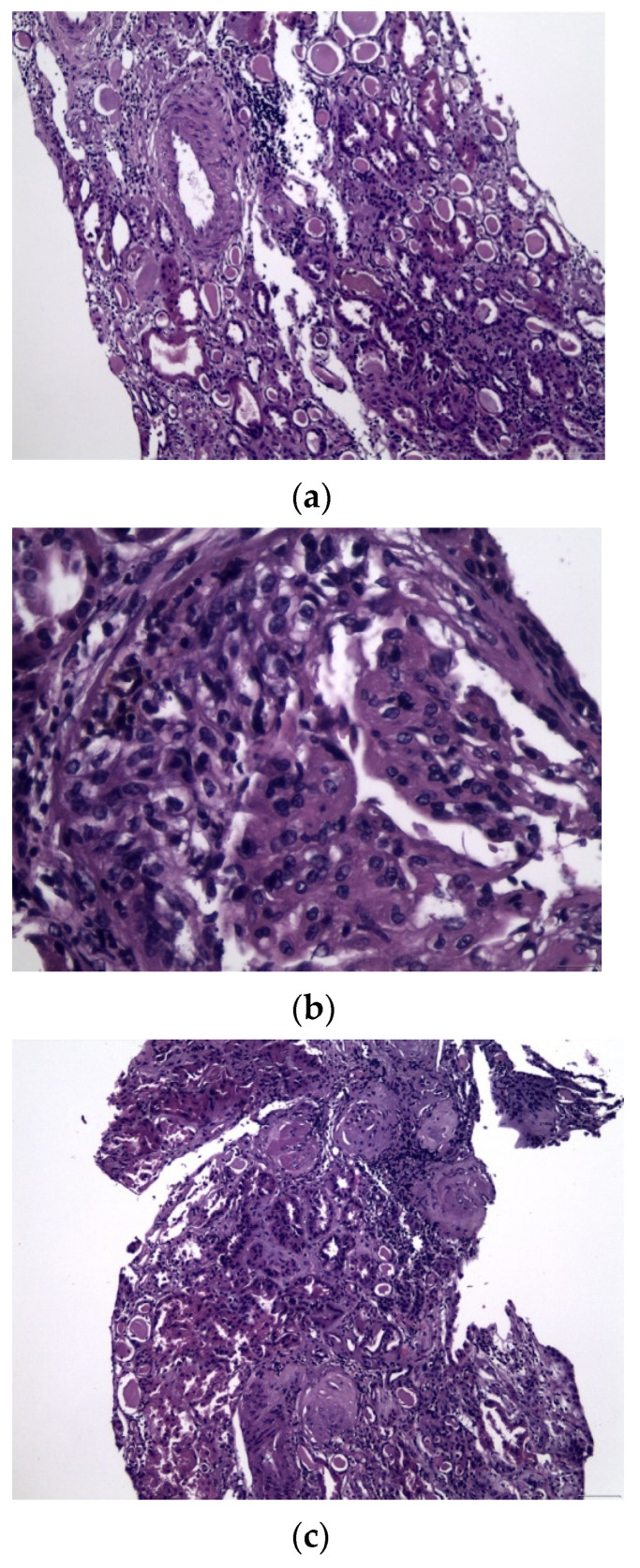
(**a**–**c**) Pauci-immune crescentic sclerotic glomerulonephritis: (**a**) focus of thyroidization of renal parenchyma; (**b**) glomerulus with a large cellular crescent; (**c**) globally sclerosed glomeruli in a moderate-to-severe fibrotic interstitium.

Serological testing revealed positive anti-MPO–ANCA IgG antibodies. Screening for anti-glomerular basement membrane autoantibodies was negative. Imaging testing with a chest computed tomography showed ground glass pulmonary infiltrates bilaterally, while flexible bronchoscopy with sequential bronchoalveolar lavage revealed 78% of macrophages stain was positive for hemosiderin, pointing to the diagnosis of recent diffuse alveolar hemorrhage in the absence of hemoptysis. A kidney biopsy was performed, which included 32 glomeruli in total (Figure 2). There was extensive global and focal glomerulosclerosis (71%), moderate interstitial fibrosis, and tubular atrophy (35%) in the absence of Kimmelstiel Wilson nodules. In total, 24 glomeruli (75%) had crescents, either cellular or fibrotic. Only one normal glomerulus was identified (Figure 2). Immunofluorescence revealed few immune deposits in the glomeruli (1+). Taking into account both serological and histopathological findings, the diagnosis of pauci-immune crescentic sclerotic MPO–ANCA GN was determined [9]. The clinical phenotype was determined as granulomatosis with polyangiitis (GPA). The Birmingham Vasculitis Activity Score was 31 at presentation [10,11,12]. 

Induction immunosuppressive therapy was administered immediately, mainly due to serious extrarenal manifestations, i.e pulmonary hemorrhage, and included intravenous pulses of methylprednisolone (500 mg daily for 3 days), followed by daily oral methylprednisolone (40 mg/day) and intravenous pulses of cyclophosphamide monthly (500 mg/m^2^ body surface area), two in total. However, two months after treatment initiation, the patient developed severe oral mucositis, with painful aphthous-like ulcerations consistent with herpes simplex virus types 1 and 2. Symptoms were eliminated after treatment with intravenously acyclovir and fluconazole intravenously. At the same time, she reported severe unilateral low back pain, and a diagnosis of recent non-traumatic lumbar fracture of the L1 vertebral body was made, which was associated with osteopenia, as confirmed with a bone mass density test. During these two months, laboratory tests revealed no improvement in renal function (serum creatinine 5.1 mg/dL, serum urea 253 mg/dL, estimated GFR 8 mL/min/1.73 m^2^, proteinuria 1.5 g/24 h, and microscopic glomerular hematuria 25–30 RBC/hpf). The patient managed to avoid life-threatening phenomena i.e massive pulmonary hemorrhage and death; however, she was unresponsive in terms of ANCA GN, probably as a resylt of the extended irreversible renal lesions noted in the diagnostic kidney biopsy. Eventually, this lead to the initiation of renal replacement therapy, while inductive therapy with cyclophosphamide was discontinued and oral methylprednisolone was gradually tapered. A rituximab-based regimen was initiated as maintenance therapy (pulses of 500 mg each) according to the MAINRITSAN scheme [13]. A reduced dose of entecavir was given prior to initiation of rituximab due to a history of HBV infection, in order to decrease the risk of reactivation. After nine months of follow-up (three administered doses of rituximab so far), she remains in complete remission of vasculitis, on dialysis with full recovery of mouth ulcers with normal mobilization. 

## 3. Discussion

In this report, we described a patient with MPO–ANCA GPA, who presented with rapidly progressive GN and extra-renal vasculitis of the skin, and upper and lower respiratory system. Although she received the standard of care, she did not recover renal function but avoided life-threatening phenomena and death. Granulomatosis with polyangiitis, microscopic polyangiitis, and eosinophilic granulomatosis with polyangiitis are different clinical phenotypes of necrotizing vasculitis, predominantly affecting small-sized vessels. Typically, there is a strong association with ANCAs [14], which are found positive in over 85% of patients. Typically, PR3–ANCA is most commonly associated with GPA (75%), whereas MPO–ANCA is more commonly associated with MPA (60%) or renal-limited vasculitis (80%) [4]. Approximately 95% of patients with active GPA are ANCA-positive (75% PR3–ANCA, 20% MPO–ANCA) [4]. It is of special interest that MPO–ANCA was found positive in this case of GPA with kidney as well as upper and lower respiratory involvement. The prevalence of GPA ranges from 2.3 to 146.0 cases per million persons, with an incidence of 0.4 to 11.9 cases per million person-years [15]. There is notable geographic variation, with GPA being more common in northern Europe and Australia. Males and females are equally affected [4]. GPA usually affects renal function, as well as the upper and lower respiratory system and skin. Choice of biopsy site depends on the affected organs, while necrotizing granulomatous inflammation is the histopathologic hallmark of GPA [14].

Our patient unfortunately developed saddle-nose deformity during the acute phase of the disease, which was indicative of granuloma formation and bone destruction in the upper respiratory system. Chondritis with the destruction of the nasal cartilage and further development of a saddle-nose deformity may also be the result of other causes such as carcinoma, lymphoma, relapsing polychondritis, or trauma. Ear, nose, and throat manifestations in systemic vasculitis are much more common in patients with GPA (estimated frequency is 90% in GPA versus 35% in microscopic polyangiitis versus 50% in eosinophilic GPA) [16]. Thus, a saddle-nose deformity is strongly indicative of GPA.

The patient received the standard of care, i.e., glucocorticoids plus cyclophosphamide, since she presented with severe involvement of vital organs, i.e lungs and kidneys. She avoided further progression of pulmonary hemorrhage but she remained dialysis-dependent. This was probably related to a critical delay in disease recognition and diagnosis, which led to significant irreversible damage, i.e., glomerulosclerosis >70% at the time of the diagnostic kidney biopsy. Despite the fact that has been shown that immunosuppressive therapy is not futile, even among patients with severe renal impairment, the percentage of normal glomeruli in the diagnostic biopsy is a major determinant for renal outcome [17]. Significantly, the main prognostic indicator of dialysis dependence at entry was the percentage of fibrous crescents. There was an increased chance of being dialysis-dependent with an increased percentage of fibrous crescents, although the predictive value was moderate. The percentage of normal glomeruli, dialysis dependence at entry, intraepithelial infiltrates, and treatment arm showed a relationship with dialysis dependence at 12 months [18]. Furthermore, findings of renal pathology in this patient were in accordance with sclerotic class by the classification provided by Berden et al. [16] and a high ANCA-associated risk score (AARS), as calculated by the report by Brix et al. [17], both predicting a low probability to become dialysis independent.

Other investigators have also reported significant therapy-related mortality during the first year following diagnosis, especially in patients with low glomerular filtration rates. Furthermore, Lee et al. found that continuation of immunosuppressive therapy beyond 3–4 months in patients who remain dialysis-dependent is of no benefit, while the risk of death due to fatal infections is heavily increasing [18,19]. Cyclophosphamide is an alkylating cytotoxic agent with substantial toxicity and potentially severe short-term side effects, including bone marrow suppression, bladder toxicity, and increased susceptibility to opportunistic bacterial, fungal, and viral infections even in the absence of neutropenia. In the long term, cyclophosphamide is associated with an increased risk for hematologic malignancies, gonadal toxicity, and infertility [20], an effect that is closely linked to the total cumulative dose [21]. In addition to strategies to eliminate its toxic effects [22,23,24], a decision to discontinue therapy, if the cost–benefit requirement is not fulfilled, is crucial. Importantly, our patient developed severe oral mucositis and non-traumatic bone fracture, and the possibility to become dialysis-independent was not realistic. Therefore, cyclophosphamide was discontinued, and methyl-prednisolone was tapered. The use of plasma exchange for patients with GPA and active kidney disease remains controversial [7,25]. In this case, plasma exchange was not used, due to negative screening for anti-glomerular basement membrane autoantibodies and rapid response to cyclophosphamide and glucocorticoids. Specifically, although plasma exchange remains a therapeutic option for patients with ANCA-associated vasculitis who present with pulmonary hemorrhage and/or rapidly progressive glomerulonephritis, this patient became stable in terms of pulmonary hemorrhage soon after receiving the methylprednisolone pulses and an infusion of cyclophosphamide. At the same time, she already had significant side effects from immunosuppression, and we considered it to be very risky for this fragile patient to add immunosuppressive therapy i.e plasma exchange at that point. In addition, renal histopathology findings were disappointing and predictive of ESKD. Finally, rituximab requires several weeks in order to be effective, and thus it is not the medication of choice for ANCA-associated vasculitis presented with pulmonary hemorrhage and rapidly progressive glomerulonephritis.

## 4. Conclusions

In conclusion, we described a case of a woman with MPO–ANCA, instead of the usual PR3–ANCA, GPA who presented with rapidly progressive GN and pulmonary involvement, while she developed saddle-nose deformity, a characteristic manifestation of GPA. Despite standard of care treatment with glucocorticoids and cyclophosphamide, she did not avoid initiation of chronic dialysis, a fact that was related to the significant irreversible kidney damage she already had at the time of diagnosis. However, immunosuppressive treatment was continued since the patient had extra-renal involvement of vital organs, such as the lungs. 

## Data Availability

No new data were created or analyzed in this study. Data sharing is not applicable to this article.

## Supplementary Materials

The following are available online at https://www.mdpi.com/article/10.3390/antib11020033/s1, Figure S1: Timeline.

## References

[1] Jennette J.C. (2003). Rapidly progressive crescentic glomerulonephritis. Kidney Int..

[2] Harris A.A., Falk R.J., Jennette J.C. (1998). Crescentic glomerulonephritis with a paucity of glomerular immunoglobulin localization. Am. J. Kidney Dis..

[3] Moroni G., Ponticelli C. (2014). Rapidly progressive crescentic glomerulonephritis: Early treatment is a must. Autoimmun. Rev..

[4] Geetha D., Jefferson J.A. (2020). ANCA-Associated Vasculitis: Core Curriculum 2020. Am. J. Kidney Dis..

[5] Pagnoux C., Quéméneur T., Ninet J., Diot E., Kyndt X., de Wazières B., Reny J.L., Puéchal X., le Berruyer P.Y., Lidove O. (2015). Treatment of systemic necrotizing vasculitides in patients aged sixty-five years or older: Results of a multicenter, open-label, randomized controlled trial of corticosteroid and cyclophosphamide-based induction therapy. Arthritis Rheumatol..

[6] Stone J.H., Merkel P.A., Spiera R., Seo P., Langford C.A., Hoffman G.S., Kallenberg C.G., St Clair E.W., Turkiewicz A., Tchao N.K. (2010). Rituximab versus cyclophosphamide for ANCA-associated vasculitis. N. Engl. J. Med..

[7] Chung S.A., Langford C.A., Maz M., Abril A., Gorelik M., Guyatt G., Archer A.M., Conn D.L., Full K.A., Grayson P.C. (2021). 2021 American College of Rheumatology/Vasculitis Foundation Guideline for the Management of Antineutrophil Cytoplasmic Antibody-Associated Vasculitis. Arthritis Care Res..

[8] Haubitz M., Bohnenstengel F., Brunkhorst R., Schwab M., Hofmann U., Busse D. (2002). Cyclophosphamide pharmacokinetics and dose requirements in patients with renal insufficiency. Kidney Int..

[9] Berden A.E., Ferrario F., Hagen E.C., Jayne D.R., Jennette J.C., Joh K., Neumann I., Noël L.H., Pusey C.D., Waldherr R. (2010). Histopathologic classification of ANCA-associated glomerulonephritis. J. Am. Soc. Nephrol..

[10] Luqmani R.A., Bacon P.A., Moots R.J., Janssen B.A., Pall A., Emery P., Savage C., Adu D. (1994). Birmingham Vasculitis Activity Score (BVAS) in systemic necrotizing vasculitis. QJM.

[11] Stone J.H., Hoffman G.S., Merkel P.A., Min Y.I., Uhlfelder M.L., Hellmann D.B., Specks U., Allen N.B., Davis J.C., Spiera R.F. (2001). A disease-specific activity index for Wegener’s granulomatosis: Modification of the Birmingham Vasculitis Activity Score. International Network for the Study of the Systemic Vasculitides (INSSYS). Arthritis Rheum..

[12] Suppiah R., Mukhtyar C., Flossmann O., Alberici F., Baslund B., Batra R., Brown D., Holle J., Hruskova Z., Jayne D.R. (2011). A cross-sectional study of the Birmingham Vasculitis Activity Score version 3 in systemic vasculitis. Rheumatology.

[13] Guillevin L., Pagnoux C., Karras A., Khouatra C., Aumaître O., Cohen P., Maurier F., Decaux O., Ninet J., Gobert P. (2014). Rituximab versus azathioprine for maintenance in ANCA-associated vasculitis. N. Engl. J. Med..

[14] Lionaki S., Blyth E.R., Hogan S.L., Hu Y., Senior B.A., Jennette C.E., Nachman P.H., Jennette J.C., Falk R.J. (2012). Classification of antineutrophil cytoplasmic autoantibody vasculitides: The role of antineutrophil cytoplasmic autoantibody specificity for myeloperoxidase or proteinase 3 in disease recognition and prognosis. Arthritis Rheum..

[15] Kitching A.R., Anders H.J., Basu N., Brouwer E., Gordon J., Jayne D.R., Kullman J., Lyons P.A., Merkel P.A., Savage C.O.S. (2020). ANCA-associated vasculitis. Nat. Rev. Dis. Primers.

[16] Jennette J.C., Falk R.J. (1997). Small-vessel vasculitis. N. Engl. J. Med..

[17] de Lind van Wijngaarden R.A., Hauer H.A., Wolterbeek R., Jayne D.R., Gaskin G., Rasmussen N., Noël L.H., Ferrario F., Waldherr R., Bruijn J.A. (2007). Chances of renal recovery for dialysis-dependent ANCA-associated glomerulonephritis. J. Am. Soc. Nephrol..

[18] Brix S.R., Noriega M., Tennstedt P., Vettorazzi E., Busch M., Nitschke M., Jabs W.J., Özcan F., Wendt R., Hausberg M. (2018). Development and validation of a renal risk score in ANCA-associated glomerulonephritis. Kidney Int..

[19] Lee T., Gasim A., Derebail V.K., Chung Y., McGregor J.G., Lionaki S., Poulton C.J., Hogan S.L., Jennette J.C., Falk R.J. (2014). Predictors of treatment outcomes in ANCA-associated vasculitis with severe kidney failure. Clin. J. Am. Soc. Nephrol..

[20] van Wijngaarden R.A.D.L., Hauer H.A., Wolterbeek R., Jayne D.R., Gaskin G., Rasmussen N., Noël L.H., Ferrario F., Waldherr R., Hagen E.C. (2006). Clinical and histologic determinants of renal outcome in ANCA-associated vasculitis: A prospective analysis of 100 patients with severe renal involvement. J. Am. Soc. Nephrol..

[21] de Jonge M.E., Huitema A.D., Rodenhuis S., Beijnen J.H. (2005). Clinical pharmacokinetics of cyclophosphamide. Clin. Pharmacokinet..

[22] Hoffman G.S., Kerr G.S., Leavitt R.Y., Hallahan C.W., Lebovics R.S., Travis W.D., Rottem M., Fauci A.S. (1992). Wegener granulomatosis: An analysis of 158 patients. Ann. Intern. Med..

[23] Jayne D., Rasmussen N., Andrassy K., Bacon P., Tervaert J.W.C., Dadoniené J., Ekstrand A., Gaskin G., Gregorini G., de Groot K. (2003). A randomized trial of maintenance therapy for vasculitis associated with antineutrophil cytoplasmic autoantibodies. N. Engl. J. Med..

[24] de Groot K., Harper L., Jayne D.R., Flores Suarez L.F., Gregorini G., Gross W.L., Luqmani R., Pusey C.D., Rasmussen N., Sinico R.A. (2009). Pulse versus daily oral cyclophosphamide for induction of remission in antineutrophil cytoplasmic antibody-associated vasculitis: A randomized trial. Ann. Intern. Med..

[25] Rovin B.H., Adler S.G., Barratt J., Bridoux F., Burdge K.A., Chan T.M., Cook H.T., Fervenza F.C., Gibson K.L., Glassock R.J. (2021). Executive summary of the KDIGO 2021 Guideline for the Management of Glomerular Diseases. Kidney Int..

