# Supplementary material for: MPO–ANCA-Positive Granulomatosis with Polyangiitis with Rapidly Progressive Glomerulonephritis and Saddle-Nose Deformity: A Case Report
Source: Antibodies (Basel). 2022 May 9;11(2):33. doi: 10.3390/antib11020033 (PMC9149920; doi:10.3390/antib11020033)
Supplement: Supplementary file 1 [file antibodies-11-00033-s001.zip › antibodies-1636849-supplementary.pdf]

antibodies

MDPI

Supplementary material

MPO–ANCA-Positive Granulomatosis with Polyangiitis with Rapidly Progressive Glomerulonephritis and Saddle-Nose Deformity: A Case Report

Dimitra Petrou [1], Minas Karagiannis [1], Petros Nikolopoulos [1], George Liapis [2] and Sophia Lionaki [1,*]

[1] Department of Nephrology, National and Kapodistrian University of Athens, Attikon Hospital, 12462 Athens, Greece; dimitra.petrou90@gmail.com (D.P.); minaskar64@gmail.com (M.K.); nikolopoulospetros@gmail.com (P.N.)
[2] Department of Pathology, National and Kapodistrian University of Athens, Laiko Hospital, 11527 Athens, Greece; gliapis@gmail.com
* Correspondence: sophial@med.uoa.gr

Citation: Petrou, D.; Karagiannis, M.; Nikolopoulos, P.; Liapis, G.; Lionaki, S. MPO–ANCA-Positive Granulomatosis with Polyangiitis with Rapidly Progressive Glomerulonephritis and Saddle-Nose Deformity: A Case Report. *Antibodies* **2022**, *11*, 33. https://doi.org/10.3390/antib11020033

Academic Editor: Loredana Frasca

Received: 27 February 2022
Accepted: 1 May 2022
Published: 9 May 2022

Publisher's Note: MDPI stays neutral with regard to jurisdictional claims in published maps and institutional affiliations.

Copyright: © 2022 by the authors. Licensee MDPI, Basel, Switzerland. This article is an open access article distributed under the terms and conditions of the Creative Commons Attribution (CC BY) license (https://creativecommons.org/licenses/by/4.0/).

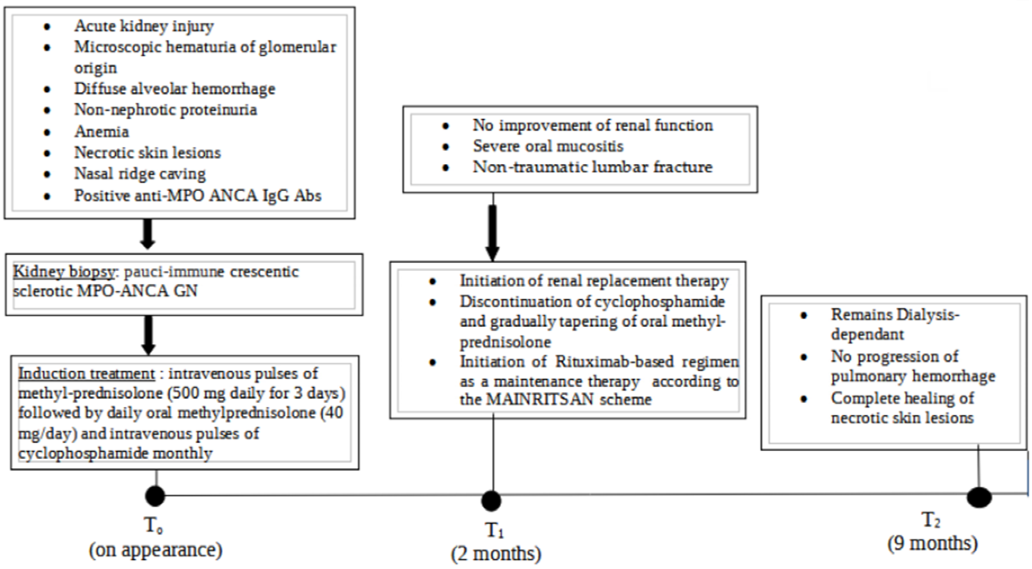

Figure S1. Timeline.
